# The Effect of Limited Diffusion and Wet–Dry Cycling on Reversible Polymerization Reactions: Implications for Prebiotic Synthesis of Nucleic Acids

**DOI:** 10.3390/life6020024

**Published:** 2016-06-08

**Authors:** Paul G. Higgs

**Affiliations:** Origins Institute & Department of Physics and Astronomy, McMaster University, Hamilton, ON L8S 4M1, Canada; higgsp@mcmaster.ca; Tel.: +1-905-525-9140

**Keywords:** origin of life, RNA world, polymerization kinetics, wetting and drying cycles, diffusion, computer simulation

## Abstract

A long-standing problem for the origins of life is that polymerization of many biopolymers, including nucleic acids and peptides, is thermodynamically unfavourable in aqueous solution. If bond making and breaking is reversible, monomers and very short oligomers predominate. Recent experiments have shown that wetting and drying cycles can overcome this problem and drive the formation of longer polymers. In the dry phase, bond formation is favourable, but diffusion is restricted, and bonds only form between monomers that are initially close together. In the wet phase, some of the bonds are hydrolyzed. However, repositioning of the molecules allows new bonds to form in the next dry phase, leading to an increase in mean polymer length. Here, we consider a simple theoretical model that explains the effect of cycling. There is an equilibrium length distribution with a high mean length that could be achieved if diffusion occurred freely in the dry phase. This equilibrium is inaccessible without diffusion. A single dry cycle without diffusion leads to mean lengths much shorter than this. Repeated cycling leads to a significant increase in polymerization relative to a single cycle. In the most favourable case, cycling leads to the same equilibrium length distribution as would be achieved if free diffusion were possible in the dry phase. These results support the RNA World scenario by explaining a potential route to synthesis of long RNAs; however, they also imply that cycling would be beneficial to the synthesis of other kinds of polymers, including peptides, where bond formation involves a condensation reaction.

## 1. Introduction

Life on Earth depends on DNA, RNA and proteins. All of these polymers form by condensation reactions that are thermodynamically unfavourable in aqueous conditions. Left to their own devices, these polymers would hydrolyze back to an equilibrium distribution of mostly monomers and a few short oligomers. Living organisms can drive synthesis of long strands through metabolism. However, the first replicating molecules must have been formed abiotically, so there must have been some environment where the chemical synthesis of fairly long biopolymers was possible.

According to the RNA World hypothesis for the Origin of Life, the first replicating molecules were nucleic acids (or some very similar kind of polymer) that had the ability to act as both a gene and a catalyst [1,2]. This is supported by the fact that catalytic RNAs (ribozymes) have been developed in the laboratory [3,4,5], and that the catalytic core of the ribosome, which synthesizes proteins in all cells today, is made of RNA [6]. If a diverse mixture of random RNAs can be synthesized abiotically, it is possible for functional ribozymes to emerge and be selected from this mixture, as we have shown using computational models [7,8,9,10,11]. However, the RNA World theory only makes sense if the means of chemical synthesis can be understood experimentally.

There has been recent progress with synthesis of individual nucleotides and with polymerization of nucleotides to form RNA strands, although neither of these problems is fully solved [12]. Here we focus on the polymerization step. One of the most widely studied methods for synthesizing RNAs uses montmorillonite clay as a catalyst [13,14]. However, it has recently been suggested that the strand lengths of up to 50 that were claimed with this method may be an over-estimate due to problems of interpreting mass spectrometry results [15]. Furthermore, these experiments use imidazole-activated nucleotides, which may not be very likely prebiotically, although alternative methods of imidazole activation have also been investigated [16]. Polymerization of imidazole-activated nucleotides has also been observed in ice eutectics [17].

Alternative experimental methods to synthesize RNA strands from non-activated nucleotides rely on finding environments that have low water activity, in which case the polymerization reaction can become thermodynamically favourable. Drying of solutions of 2′,3′-cAMP can drive formation of short oligomers, as was shown some time ago [18]. More recently, however, it has become apparent that cycling wet and dry conditions gives a significant improvement in the degree of polymerization with respect to simply drying. Wet–dry cycles in the presence of phospholipids can lead to RNA-like polymers in the 25–75 nucleotide range, and sometimes up to 100 [19,20]. It has also been shown that wetting and drying cycles of 5′ monophosphate nucleotides in the presence of salts such as ammonium chloride leads to a broad range of lengths up to as much as 300-mers [21]. The likelihood of polymer bond formation between nucleotides should depend on the relative positioning of the nucleotides as they are brought together by the drying procedure. X-ray scattering has been used to look at the arrangement of nucleotides that are confined between lipid bilayers [22] and also captured in matrices of ammonium chloride and montmorillonite clay [23]. The lipids, salt, and clay seem to share the feature that they allow nucleotides to come together in a ‘pre-polymer’ arrangement in which the alignment is favourable for bond formation.

The dynamics of these experiments is important, not just the thermodynamics. If only thermodynamics were relevant, the best thing to do would be to remain in the dry phase without cycling. In fact, however, although dry conditions favour polymerization, dry conditions do not usually permit diffusion, or at least, diffusion is likely to be much slower than in solution. Monomers may only link together if they encounter one another; hence only monomers that are very close after the drying procedure are able to polymerize. The likely benefit of the wet phases is that they permit rearrangement of molecules and bring molecules together that may polymerize in the next dry phase. Wet phases inevitably permit hydrolysis and act against the polymerization that occurs in the dry phases. However, if the wet phase is relatively short, then the net effect of cycling may be an increase in polymerization relative to a single dry phase with no cycling. The main aim of this paper is to understand when and why cycling can lead to an increase in polymerization.

The theory in this paper applies to formation of any kind of polymer where there is a condensation reaction that is thermodynamically favorable in the dry phase. Thus, in addition to applying to nucleic acid synthesis in an RNA World scenario, it would also apply to peptide synthesis, for example. It has been shown experimentally that cycling is also relevant for other kinds of biopolymers, such as depsipeptides [24]. An experiment with malic acid polymers shows that wet–dry cycles cause a ratcheting up of the polymer length [25]. These results were compared to a predictions of a simple chemical reaction model that describes reversible polymerization and hydrolysis. However, the model considered in ref [25] does not describe the diffusion of the molecules, or the lack of diffusion in the dry phase; hence it cannot explain why cycling is beneficial with respect to a single dry phase.

Here, we give a theory that clearly explains the interplay between diffusion, wet–dry cycles, and reversible polymerization. Initially, we consider a model where only dimers are permitted. This model is simple enough to solve exactly, and hence gives a straightforward proof of the effect. We show that if diffusion is possible during the dry phase, an equilibrium is reached with a relatively high concentration of dimers. If diffusion cannot occur in the dry phase, this equilibrium cannot be achieved, and the system is stuck in a state with fewer dimers. Wet–dry cycling leads to a significant improvement over this restricted state.

We then consider a model for formation of polymers of all lengths. In the polymer case, the limiting equilibrium state can be calculated, and the dynamics of the model can be studied by Monte Carlo simulation. We show that repeated wet–dry cycling leads to a significant increase in mean polymer length relative to the case of a single dry cycle. In the optimal case for cycling, where hydrolysis in the wet phase is negligible, the same equilibrium is reached as would occur if diffusion were possible in the dry phase. Hence, cycling allows the formation of long polymers in conditions where thermodynamics favours polymerization in the dry phase but lack of diffusion prevents this polymerization from occurring in absence of cycling.

Finally, we consider a case where continued influx of new monomers is permitted during the cycling procedure. This leads to a further increase in the benefit of cycling relative to the case in which the total number of monomers is fixed.

## 2. Materials and Methods

We use analytical theory where possible and supplement this with Monte Carlo simulations where an exact solution is not possible. Details of the simulations are given at the appropriate point in the Results section.

## 3. Results

### 3.1. Dimer Formation Model

Here we describe a model of dimer formation that illustrates the effects of limited diffusion and wetting and drying cycles in a simple way that is exactly solvable analytically.

**Figure 1:**
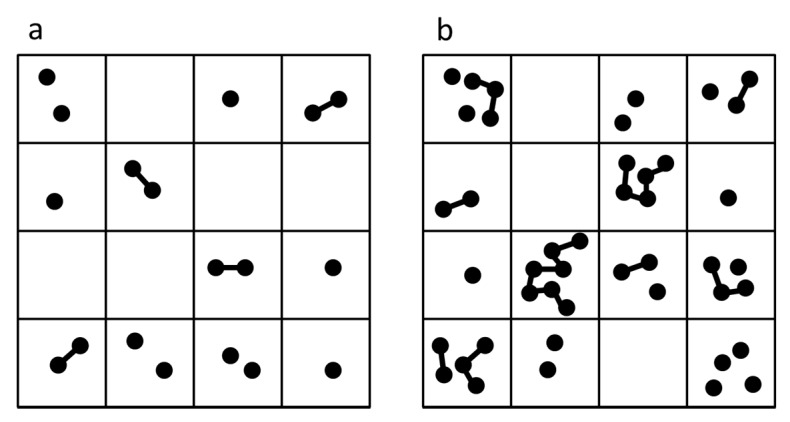
(**a**) A typical lattice configuration in the dimer model. A site may have either zero, one or two monomers, or one dimer; (**b**) A typical lattice configuration in the polymer model. Strands of all lengths are permitted. There is no constraint on the number of monomers and strands on any one site, but the total number of monomers in the whole lattice is fixed.

The dry phase is represented by a 2d lattice where each site may contain 0, 1 or 2 monomers (see Figure 1a). Two monomers on one site can react to form a dimer at rate $k^{+}$. Dimers can break at rate $k^{-}$. The equilibrium constant for dimer formation is $K=k^{+}/k^{-}=\exp(-\Delta G/RT)$, where negative ΔG corresponds to a favourable free energy of bond formation (*K* > 1). If diffusion is possible in the dry phase, monomers and dimers are allowed to move into vacancies on neighbouring sites, as described further below. The wet phase is assumed to be dilute, so that no further bond formation occurs, but breakup of dimers occurs at rate *w*. The spatial position of molecules is not relevant in the wet phase. At the end of the wet phase, each molecule is placed down in a new random position on the lattice, and a further dry phase begins. We consider three cases: (i) a single dry phase in which diffusion is permitted; (ii) a single dry phase with no diffusion; (iii) cycling between dry phases with no diffusion and wet phases with random repositioning of molecules.

#### 3.1.1. Case 1—Dry Phase with Diffusion Permitted

If the lattice has *N* sites, the maximum occupancy of monomers is 2*N*. The total number of monomers is *M_tot_*, which is assumed to be fixed. The overall concentration of monomers is $\phi =M_{tot}/2N$, relative to the maximum occupancy. Let the fractions of sites with 0 monomers, 1 monomer, 2 monomers and 1 dimer be *P_0_*, *P_1_*, *P_2_*, and *P_D_*. Initially, the monomers are placed down at random on the lattice, with no dimers present; therefore, to begin with

(1) $$P_{0}={\left(1-\phi \right)}^{2};P_{1}=2\phi (1-\phi );P_{2}=\phi^{2};P_{D}=0$$

Monomers attempt to move to another site at rate *h*. One of the eight neighbouring sites is chosen at random. If the neighbouring site is empty, the move succeeds. If the site has one monomer, the move succeeds half the time. If the site has two monomers or a dimer, the move fails because the site is occupied. Dimers can attempt to move to a neighbouring site at rate *h_D_*. This only succeeds if the site is empty.

We will now solve this model in the mean field approximation, where we assume there is no correlation between the states of neighbouring sites. This will be true if *h* is large, or if hopping occurs to a random site, rather than only to a neighbouring site. In the mean field case, the only hopping terms that are relevant are of the form 2 + 0 ↔ 1 + 1, *i.e.*, a monomer moves from a site with two monomers to an empty site, creating two sites with one monomer, or the reverse of this, where a monomer moves from a site with one monomer to another site with one monomer, creating a site with two monomers and an empty site. There are three other processes that exchange molecules between sites: 1 + 0 ↔ 0 + 1; 2 + 1 ↔ 1 + 2; and D + 0 ↔ 0 + D. However, the other processes do not change the numbers of sites of each kind, and so they do not appear in the theory. As the dimer hopping process does not change the numbers of sites, it turns out that *h_D_* is not relevant in the mean field theory. The differential equations for the probabilities of the four types of site are:

(2) $$\frac{dP_{0}}{dt}=-2hP_{0}P_{2}+\frac{h}{2}P_{1}^{2}$$

(3) $$\frac{dP_{1}}{dt}=4hP_{0}P_{2}-hP_{1}^{2}$$

(4) $$\frac{dP_{2}}{dt}=-2hP_{0}P_{2}+\frac{h}{2}P_{1}^{2}-k^{+}P_{2}+k^{-}P_{D}$$

(5) $$\frac{dP_{D}}{dt}=k^{+}P_{2}-k^{-}P_{D}$$

The following conditions apply at equilibrium:

Constant fraction of vacancies:

(6) $$1-\phi =P_{0}+\frac{1}{2}P_{1}$$

Constant fraction of occupied sites:

(7) $$\phi =\frac{1}{2}P_{1}+P_{2}+P_{D}$$

Balance of dimer making and breaking:

(8) $$P_{D}=\frac{k^{+}}{k^{-}}P_{2}=KP_{2}$$

Balance of diffusion:

(9) $$4P_{0}P_{2}=P_{1}^{2}$$

Using Equations (6)–(8), all probabilities can be expressed in terms of *P_1_*. Hence, from Equation (9),

(10) $$KP_{1}^{2}+2P_{1}-4\phi (1-\phi )=0$$

The relevant solution is:

(11) $$P_{1}=\frac{1}{K}(-1+\sqrt{1+4K\phi (1-\phi )})$$

(12) $$P_{2}=\frac{1}{2K(1+K)}\left(2K\phi +1-\sqrt{1+4K\phi (1-\phi )}\right)$$

(13) $$P_{D}=\frac{1}{2(1+K)}\left(2K\phi +1-\sqrt{1+4K\phi (1-\phi )}\right)$$

In the limit of *K* → 0, there is no dimer formation and the site probabilities are the same as the initial conditions. In the limit of *K* >> 1, all the monomers are converted to dimers; therefore *P_1_ = P_2_ =* 0, and *P_D_* = *φ*.

#### 3.1.2. Case 2—Dry Phase without Diffusion

In this case, we start with randomly positioned monomers, as in Equation (1), but no diffusion is permitted. Dimers can only form on sites where there are initially two monomers. The sum $P_{2}+P_{D}=\phi^{2}$ is constant because there is no diffusion.

(14) $$\frac{dP_{D}}{dt}=k^{+}P_{2}-k^{-}P_{D}=k^{+}\phi^{2}-(k^{+}+k^{-})P_{D}$$

At local equilibrium (without diffusion)

(15) $$P_{D}=\frac{K}{1+K}\phi^{2}$$

**Figure 2:**
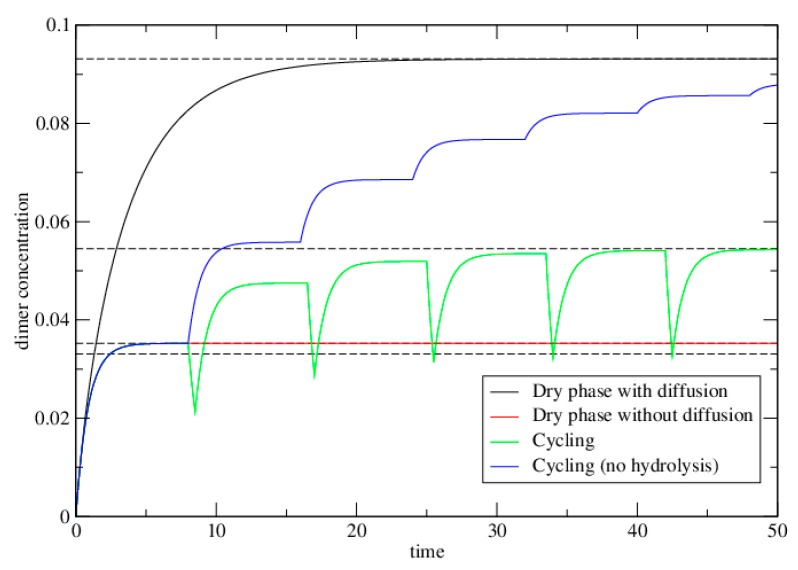
The dimer concentration, *P_D_*, as a function of time in the dimer-formation model. Dashed lines show theoretical limits that are calculated in the text. Solid lines show *P_D_* as a function of time obtained by numerical solutions of the differential Equations (2)–(5). Parameters are *φ =* 0.2, *k^+^* = 1, *k^−^* = exp(−2), and *K =* exp(2). In the diffusion case, *h* = 1. In the cycling case, *w* = 1, *t_dry_* = 8, and *t_wet_* = 0.5. In the case with no hydrolysis, *t_dry_* = 8, and *t_wet_* = 0. This latter case reaches the same limit as the diffusion case after many cycles. Time units are arbitrary, but defined relative to the rate *k^+^* = 1.

In the high *K* limit, the maximum dimer concentration is *φ^2^*, which is always less than the maximum concentration *φ* that is achievable when diffusion occurs. Figure 2 compares cases 1 and 2 for a moderate value of *K* = exp(2), which corresponds to a free energy of bond formation Δ*G* = −2*RT*. It can be seen that if diffusion is not possible in the dry phase (red curve), the dimer concentration remains much lower than it would be if diffusion were possible (black curve).

#### 3.1.3. Case 3—Alternating Dry and Wet Cycles

In the cycling case, let *t_dry_* and *t_wet_* be the length of the two phases. The first dry phase is the same as case 2. Subsequent dry phases are similar to case 2, but have different initial conditions. Let *N_D_* be the number of dimers at the end of a wet phase. The number of monomers is $M=M_{tot}-2N_{D}$. At the beginning of the dry phase the dimers are placed down randomly on the lattice. The initial fraction of dimer sites is $P_{D}^{init}=N_{D}/N$. The *M* monomers are then distributed among the remaining *N* − *N_D_* sites. The concentration on these sites is

(16) $$\xi =\frac{\left(M-2N_{D}\right)}{2(N-N_{D})}=\frac{\phi -P_{D}^{init}}{1-P_{D}^{init}}$$

Therefore the fraction of sites with two monomers is

(17) $$P_{2}^{init}=(1-P_{D}^{init})\xi^{2}=\frac{{\left(\phi -P_{D}^{init}\right)}^{2}}{1-P_{D}^{init}}$$

During the dry phase with no diffusion, *P_D_* follows Equation (14). If the dry phase is long enough to reach local equilibrium, then the fraction of sites with dimers at the end of the dry phase is

(18) $$P_{D}^{dry}=\frac{K}{1+K}\left(P_{D}^{init}+\frac{{\left(\phi -P_{D}^{init}\right)}^{2}}{1-P_{D}^{init}}\right)$$

and the number of dimers at this point is $N_{D}^{dry}=NP_{D}^{dry}$. In the wet phase, breakup of dimers occurs at rate *w*, and there is no further bond formation. After a time *t_wet_* the number of remaining dimers is $N_{D}^{wet}=NP_{D}^{dry}\exp(-wt_{wet})$. The lattice sites are not relevant in the wet phase, but it is useful to keep the same concentration scale by defining $P_{D}^{wet}=N_{D}^{wet}/N$. At the end of this wet phase, the molecules are put down again on the lattice for the beginning of the next dry phase. After many cycles, the system will reach a limit state where $P_{D}^{wet}=P_{D}^{init}$. If we define $f=\frac{K\exp(-wt_{wet})}{1+K}$, the limit value of $P_{D}^{wet}$ satisfies

(19) $$f\left(P_{D}^{wet}+\frac{{\left(2\phi -2P_{D}^{wet}\right)}^{2}}{4(1-P_{D}^{wet})}\right)=P_{D}^{wet}$$

Rearranging this gives a quadratic equation for which the solution is

(20) $$P_{D}^{wet}=\frac{1}{2}\left(1-f+2f\phi -\sqrt{{\left(1-f+2f\phi \right)}^{2}-4f\phi^{2}}\right)$$

Note that this is the lower limit of the concentration under repeated cycles (at the end of the wet phase). The upper limit under repeated cycles (at the end of the dry phase) is $P_{D}^{dry}=P_{D}^{wet}\exp(wt_{wet})$. The upper and lower limits under cycling are shown as dashed lines on Figure 2. These limits are reached after about six cycles for the parameters illustrated.

The highest concentration of dimers is achieved when the wet phase is short, *wt_wet_* << 1 and *f = K/*(1 + *K*). Substituting this value of *f* into equation 20 shows that the limiting *P_D_* under cycling becomes equal to the limiting *P_D_* when diffusion is allowed (equation 13). This model clearly shows that best case for dimerization is the single dry phase with diffusion. Cycling can never do better than this limit, but can approach this limit if *wt_wet_* << 1. The upper limit under cycling is considerably better than the local equilibrium achieved by a single dry cycle without diffusion, even if there is a non-negligible degree of hydrolysis in the wet phase.

### 3.2. Polymerization Model

#### 3.2.1. Well-Mixed Case

The simplest model for reversible polymerization is the well-mixed case where polymers encounter one another in proportion to their concentration. In this case, the equilibrium concentration $C_{m}$of polymers of length *m* is exponentially decreasing

(21) $$C_{m}=ax^{m-1}$$

for some *a* and *x*, which can be calculated as follows. Consider the formation of a chain of length *n* + *m* by linking chains of length *n* and *m* and the corresponding hydrolysis by breaking the long chain at the corresponding point. The equilibrium condition is

(22) $$k^{+}C_{n}C_{m}=k^{-}C_{n+m}$$

This is satisfied if *x = aK*, where $K=k^{+}/k^{-}$. The exponential distribution (Equation (21)) satisfies this equilibrium condition for all values of *n* and *m*. If the total concentration *μ* of monomers in the system is fixed, then

(23) $$\sum_{m=1}^{\infty }mC_{m}=\frac{a}{{\left(1-x\right)}^{2}}=\mu$$

Using the fact that *a = x/K* gives a quadratic equation ${\left(1-x\right)}^{2}=x/K\mu$, from which

(24) $$x=1+\frac{1}{2K\mu }-\sqrt{{\left(1+\frac{1}{2K\mu }\right)}^{2}-1}$$

All the concentrations are now known as a function of *K* and *μ*. If bond formation is thermodynamically favourable, then Δ*G* < 0, and *K >* 1. In this case, if we begin with a high monomer concentration *μ*, bond formation will initially be faster than breaking, and the mean length of polymers will grow. However, once we reach equilibrium, the concentration of free monomers *a* will be much less than *μ*, and there will be a balance between bond formation and breaking. At equilibrium, *x* is always less than 1, which means that the polymer concentration always *decreases* as a function of length, even when *K* > 1.

The mean length of a polymer is

(25) $$\bar{m}=\frac{\sum_{m}mC_{m}}{\sum_{m}C_{m}}=\frac{\mu }{C_{tot}}$$

where *μ* is the total number of monomers per unit volume (which is fixed) and *C_tot_* is the total number of strands per unit volume (which varies as the polymers grow and break up). At equilibrium, $\bar{m}=1/(1-x)$ from Equation (21). For *K* >> 1, *x* approaches 1, therefore the mean length can become very long when the free energy of bond formation is very negative.

It is useful to consider the way the mean length changes with time as it approaches equilibrium. In the well-mixed case there is a straightforward reaction kinetics equation for *C_tot_*. For every strand, there is one less hydrolysable bond than the number of monomers. Therefore, the concentration of hydrolysable bonds is *μ* − *C_tot_*. Hence,

(26) $$\frac{dC_{tot}}{dt}=-k^{+}C_{tot}^{2}+k^{-}(\mu -C_{tot})$$

**Figure 3:**
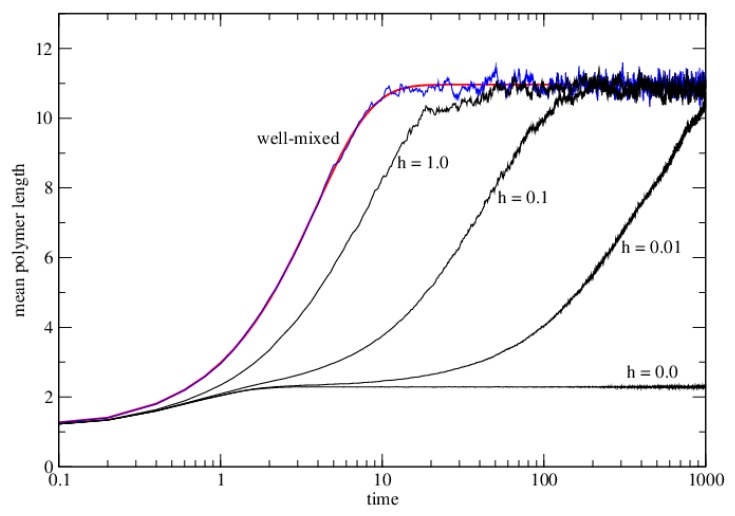
The mean polymer length (in monomers) as a function of time in the lattice model for polymerization with several values of hopping rate *h* (black curves) and for the scrambling case, corresponding to *h >>* 1 (blue curve). The smooth red curve is calculated from reaction kinetics equations for the well-mixed case. Parameters are *μ =* 2, *k^+^* = 1, *k^−^* = exp(−4), and *K =* exp(4). The time scale is logarithmic because the time taken to reach equilibrium is much longer when *h* is small. The case with no diffusion (*h =* 0) never reaches the equilibrium.

*C_tot_* can be followed as a function of time by numerical integration of this equation, and from this we know the mean length as a function of time from Equation (25). The results of this calculation will be shown below in Figure 3.

The reaction kinetics equations for concentrations for each length *C_m_* can also be written down (see, for example, [25]). In the well-mixed case it is assumed that diffusion is fast enough for all the molecules to encounter one another at a rate proportional to their concentration. We do not give details of the reaction kinetics equations here because they only apply in the well-mixed case, and not when diffusion is slow, which is the case of interest in this paper. Below, we consider a lattice model for polymerization, as this will allow us to account for the effects of limited diffusion in the dry phase.

#### 3.2.2. Lattice Model for Polymerization

Let there be a total number of monomers *M_tot_* distributed across a lattice of *N* sites. For simplicity, we make no restriction on the number of monomers that can occupy one site, although the total number of monomers remains fixed, so the mean number of monomers per site is $\mu =M_{tot}/N$. A typical configuration of the model is shown in Figure 1b. Any two strands on the same site may link at rate $k^{+}$. The hydrolysis rate is $k^{-}$ per bond in a strand. Equilibrium properties of the model can be calculated, but we do not have an exact treatment of the dynamics; therefore we use Monte Carlo simulations to follow the dynamics. In the simulations, bond formation, hydrolysis and diffusion are treated as stochastic events. In each time step of length *δt*, each site is visited once. The probability of a bond forming is $k^{+}n(n-1)\delta t$, where *n* is the number of strands on the site. If a bond forms, then two randomly-chosen strands are linked. Each strand is then considered randomly. A strand of length *m* has *m* − 1 bonds, and therefore undergoes hydrolysis with probability $k^{-}(m-1)\delta t$. If this occurs, the break point is chosen randomly along the strand. Diffusion is controlled by the hopping rate *h*. Each strand is given a probability *hδt* of hopping to one of the eight neighbouring sites. An attempted hop is always successful in this model, because no maximum limit is applied to the number of strands on one site.

We have carried out Monte Carlo simulations to illustrate the dynamics of this case. We used a square lattice of size *N* = 100^2^ = 10,000, with *M_tot_* = 20,000. Monomers are randomly positioned on lattice initially, so that *μ* = 2. We chose *K* = exp(4), meaning Δ*G* = −4*RT* for each bond formed. Figure 3 shows the mean polymer length as a function of time in the lattice model with different values of *h*. The limiting case of very fast hopping rate (*h >>* 1) can be simulated by ‘scrambling’ strand positions every time step, *i.e.*, by moving every strand to a completely random site after every time step. The lattice model with scrambling is equivalent to the well-mixed case above. The well-mixed solution is obtained by numerical integration of equation 26 and setting the mean length to be *μ/C_tot_*. The result of the lattice model with scrambling follows the well-mixed case exactly, except that there are fluctuations because of the finite number of monomers in the Monte Carlo simulation (as can be seen in Figure 3). For finite values of *h*, the mean length increases more slowly than the well-mixed case. The three curves with *h* = 1.0, 0.1 and 0.01 all tend to the same equilibrium mean length at long times; however, when diffusion is slow compared to the polymerization reaction rate (as with *h* = 0.01), the system takes very much longer to reach the equilibrium than in the well-mixed case. Reaction kinetics (Equation (26)) is a poor predictor of what happens when diffusion is slow, which is why we have used Monte Carlo simulations here.

Figure 3 also shows the result from the Monte Carlo simulation with *h =* 0. In this case there is no diffusion allowed in the dry phase (as with Case 2 in the dimer model). In this case only monomers that are initially on the same site can react. The mean length remains very low and never reaches the equilibrium that is obtained when *h* is non-zero.

We will now show that the equilibrium solution of the lattice model when diffusion is fast is the same as the well-mixed case above. We will assume that the mean number of strands of length *m* per site is $C_{m}=ax^{m-1}$, for some *a* and *x* to be calculated. The condition that the mean number of monomers per site is *μ* gives Equation (23), the same as for the well mixed case. The mean number of strands per lattice site, *λ*, is

(27) $$\sum_{m=1}^{\infty }C_{m}=\frac{a}{1-x}=\lambda$$

where *λ* is not yet known. If diffusion is fast, then strands will be distributed randomly across sites. If the total number of lattice sites and the total number of strands is large, then the probability *P*(*n*) of having *n* strands on one site is a Poisson distribution:

(28) $$P(n)=\frac{\lambda^{n}}{n!}e^{-\lambda }$$

For a site with *n* strands, there are *n*(*n* − 1) ways of joining them. The mean rate of bond formation, averaged over sites is

(29) $$k^{+}\sum_{n=2}^{{}^{\infty }}n(n-1)P(n)=k^{+}\lambda^{2}$$

For a strand of length *m* monomers, there are *m* − 1 ways of breaking it. The mean rate of hydrolysis averaged over sites is

(30) $$k^{-}\sum_{m=2}^{\infty }\left(m-1)C_{m}=k^{-}(\mu -\lambda \right)$$

Formation and breakup rates must be equal at equilibrium. Therefore

(31) $$K\lambda^{2}=\mu -\lambda$$

Substituting for *λ* and *μ* from Equations (23) and (27) into Equation (31), we obtain *x = aK*, which is the same as for the well mixed case. Hence, when diffusion is fast, we have the same solution of the lattice model as for the well-mixed case. Note also that Equation (26) reduces to equation 31 when equilibrium is reached, *i.e.*, the equilibrium value of *C_tot_* is *λ*.

#### 3.2.3. Lattice Model for Polymerization with Wet–Dry Cycling

**Figure 4:**
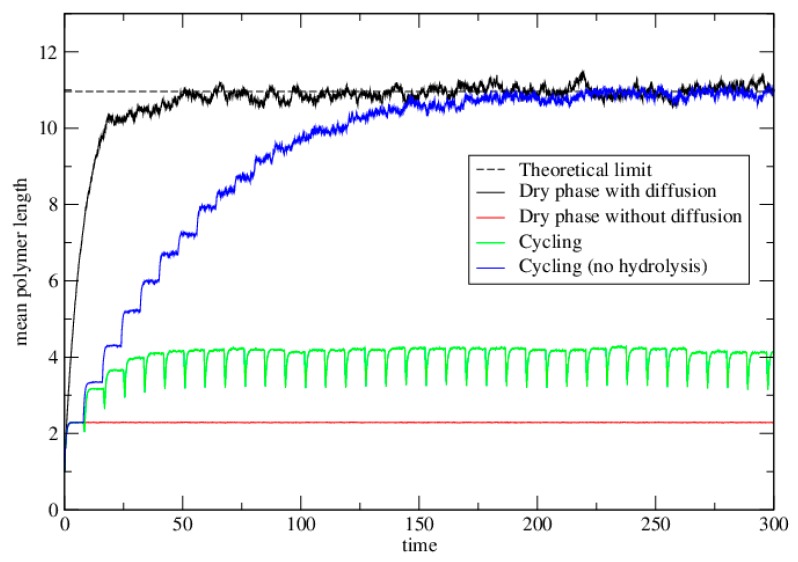
The mean polymer length as a function of time in the polymerization model. The dashed line shows the theoretical limit for the dry phase with diffusion. Solid lines show results obtained from Monte Carlo simulations. Parameters are *μ =* 2, *k^+^* = 1, *k^−^* = exp(−4), and *K =* exp(4). In the diffusion case, *h* = 1. In the cycling case, *w* = 0.2, *t_dry_* = 8, and *t_wet_* = 0.5. In the case with no hydrolysis, *t_dry_* = 8, and *t_wet_* = 0. This latter case reaches the same limit as the diffusion case after many cycles.

We also simulated the cycling case. Cycling will be most beneficial when there is no diffusion in the dry phase. Therefore we simulate the case with *h* = 0 in the dry phase. In dry phases there is bond formation and hydrolysis at rates $k^{+}$ and $k^{-}$ as before. In wet phases, there is hydrolysis at rate *w* per bond. Each strand is moved to a random new lattice site at the end of each wet phase. Figure 4 shows the case of a single dry phase with diffusion (*h =* 1) and no diffusion *(h* = 0). These are the same as the corresponding curves on Figure 3, but the time axis is linear. The cycling case is also shown (green curve). As with the dimer model, repeated cycling leads to a steady state that is intermediate between the case with diffusion in the dry phase, and without diffusion.

Finally we simulated the case where *t_wet_* = 0, so that there is no hydrolysis but the strands are randomly repositioned after each wet phase (Figure 4, blue curve). Although *t_wet_* could never be zero in an experiment, it may be the case that *wt_wet_* is very small. The point of this limiting case is to show that it tends to the same limit as the well-mixed case. Thus, cycling is an effective way to synthesize long polymers, provided the wet phase is relatively short compared to the typical time for hydrolysis in the wet phase (*wt_wet_ <* 1).

**Figure 5:**
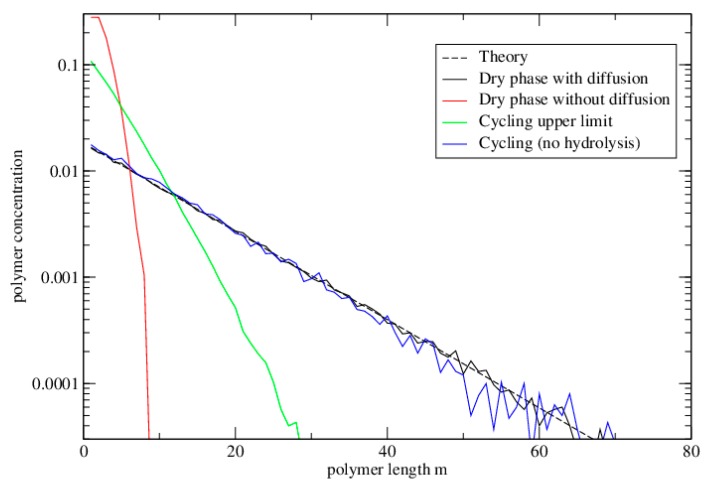
The distribution of polymer lengths in the polymerization model. Parameters are as for Figure 4. The dashed line is the theoretical equilibrium solution for the well-mixed case. The simulations for the dry phase with diffusion, and for cycling with no hydrolysis both reach this theoretical limit. The length distribution for the single cycle without diffusion falls off much more rapidly than this. The distribution at the upper limit under cycling (at the end of the dry phase) is intermediate between these.

Figure 5 shows the equilibrium distribution of lengths in these simulations. The dashed line is the theoretical equilibrium solution for the well-mixed model. The simulations for the dry phase with diffusion, and for cycling with no hydrolysis both reach this theoretical limit. The length distribution for the single cycle without diffusion falls off much more rapidly than this. The distribution at the upper limit under cycling (at the end of the dry phase) is intermediate between these.

### 3.3. Polymerization Model with Monomer Influx

A second reason why wet–dry cycling may be beneficial is that the wet phase may allow the influx of new monomers to the reacting system. We model monomer influx by allowing the mean number of monomers per site, *μ*, to increase over time according to

(32) $$\frac{d\mu }{dt}=b(1-\frac{\mu }{\mu_{\max}})$$

The parameter *b* represents a constant influx rate, and the parameter *μ_max_* represents the maximum monomer concentration that is achievable. Beginning with some initial concentration *μ_0_*, the monomer concentration will rise till it reaches *μ_max_*. This can be interpreted in several ways. Either we can say that there is no further space for adding monomers when *μ* reaches *μ_max_*, or we can say that the term *bμ/μ_max_* in Equation (32) represents flow of monomers out of the system, so that influx and efflux are balanced when *μ = μ_max_*. In the flow interpretation, we suppose that polymers are trapped in the system, and only monomers can flow in and out. A third interpretation of Equation (31) is that *b* is the rate of synthesis of monomers from precursors, and that *bμ/μ_max_* is the rate of breakdown of monomers back to precursors. Synthesis and breakdown are balanced when *μ* = *μ_max_*.

**Figure 6:**
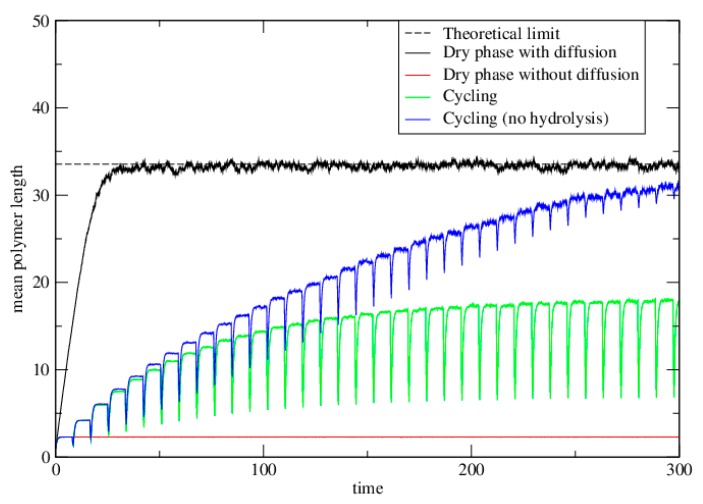
The mean polymer length as a function of time in the polymerization model with monomer influx. The initial number of monomers per site is *μ_0_* = 2. Monomers are added at rate *b* = 5, up to a maximum of *μ_max_* = 20. The other parameters are the same as Figure 4. The simulations reach much longer lengths than Figure 4 because the monomer concentration is higher, even though *K* is the same.

Initially, we consider the case where monomer influx and diffusion can both occur in the dry phase. This is the most favourable case for polymerization, although it is presumably not realizable in practice. In this case, *μ* rises till it reaches *μ_max_*, and the equilibrium solution is calculated from Equation (24) with *μ* = *μ_max_*. When simulating this model, we add monomers in random positions at each time step. The mean number of monomers added in a time step is $Nb(1-\mu /\mu_{\max})\delta t$. Figure 6 shows the result of simulating this case starting from concentration *μ_0_* = 2 (which was the fixed concentration in Figure 4) and rising to *μ_max_* = 20. The equilibrium constant for bond formation *K* is the same in both cases, but the mean polymer length becomes much longer when monomer influx occurs because higher concentration shifts the equilibrium toward longer polymers.

In practice, however, it is probable that the dry phase has no diffusion and no monomer influx. If this is the case, a single dry cycle is the same as a single dry cycle in Figure 4 with no influx. The interesting case is where we consider alternation between a dry phase with no influx and no diffusion and a wet phase with monomer influx and repositioning of all molecules at the end of the wet phase. For the simulations of this case, we keep the number of monomers in the system fixed during each dry phase, and we add monomers according to Equation (32) during the wet phases. Figure 6 shows that this results in mean polymer lengths considerably larger than the single dry phase case, and also considerably larger than the cycling case with fixed number of monomers (as in Figure 4).

The final curve (blue) in Figure 6 is the cycling case in the limit where hydrolysis is negligible in the wet phase. For this example, we keep *t_wet_* = 0.5 and set *w =* 0, so that there is a finite time for influx of monomers, but there is no hydrolysis. Once again, in this most favourable case for cycling, the limit achieved under cycling is the same as that achieved if diffusion and monomer influx could occur in the dry phase. The mean polymer length falls during the wet phases (even though *w* = 0) because we are adding new monomers in the wet phases, and these monomers contribute to the mean length calculation.

## 4. Discussion

The central message of this paper is that cycling conditions help polymerization for reasons of kinetics not equilibrium thermodynamics. If equilibrium could be achieved simply in the dry phase, there would be no point to cycling, because hydrolysis in the wet phase would always be disadvantageous. Diffusion of monomers and polymers is necessary for this equilibrium to be reached. If diffusion cannot occur in the dry phase, then inclusion of a wet phase is necessary, and the result after repeated cycles is more favourable than could be achieved with a single dry phase without diffusion. The wet phase may have other advantages that are separate from the diffusion effect. For example, if further input of monomers is linked to the wet phase, as we considered in the last section of this paper, then the wet phase is clearly beneficial. Also, we presume that functional ribozymes, if and when they emerged from the prebiotic mixture, would have worked in water. So cycling through a wet phase would also enable replicating molecules to reach an environment where they can operate. The wet phase may also involve trapping of the polymers inside lipid vesicles, which is a potential route towards formation of protocells and increasing the complexity of life [26].

For simplicity, in this paper we assumed that conditions switch instantaneously between the wet and dry states. If we included a gradual drying process, we would need extra parameters to describe the rate of drying and the way the reaction rates depend on the amount of water remaining as a function of time during the drying. This could be done, but it would not change the main conceptual point of this paper. Here, we allowed reversible polymerization in the dry phase (*i.e.*, both $k^{+}$ and $k^{-}$), and we only allowed hydrolysis (*w*) in the wet phase. Variations that could be considered would be to disallow hydrolysis completely in the dry phase ($k^{-}$ *=* 0) or to allow a non-zero rate of bond formation during the wet phase. Both of these cases would be slightly more favourable for polymerization than the case considered.

In the cycling simulations, we assumed that there is no diffusion at all in the dry phase. This was done because the maximum benefit of cycling occurs in this case. If the dry phase is completely without solvent then this seems reasonable. However, for the lipid-assisted polymerization in the lamellar phases, the lipids are a liquid crystal with some fluidity. Slow diffusion of the nucleotides between the layers may still be possible, although this would still be much slower than diffusion in solution. We showed the case of a dry phase with slow diffusion in Figure 3. In this case, the time taken to reach equilibrium is very much longer when *h* is small than when *h* is large. Adding wet cycles in the case where *h* is small but non-zero would also help to increase the rate of formation of long polymers, although the equilibrium distribution would eventually be achievable without cycling as long as *h* is non-zero. Another additional feature that could potentially be added in future is to consider the dependence of diffusion on the polymer length, with short sequence allowed to diffuse more freely than longer ones.

The importance of diffusion in models for the origin of life and replication has been noted before by several authors [27,28,29] and in our previous papers [10,11]. In all these papers, slow diffusion of replicating strands is beneficial because it allows the formation of clusters of cooperating replicators, which is an important means of resisting the invasion of parasites. However, the present paper discusses prebiotic polymerization prior to the existence of replicators, and the effect of diffusion is different here. Slow diffusion of monomers and oligomers is detrimental in the present paper because it slows down the reactions that form polymers.

In this paper we considered a polymerization reaction with a single type of monomer where the equilibrium constant for each bond formation is the same. For this reason, the equilibrium length distribution is exponential. Prevention of diffusion prevents this exponential distribution from being achieved, and leads to a length distribution that falls off much more rapidly, as was shown in Figure 4. Several other factors that we did not include in this model would affect length distributions in realistic cases. If monomers of different types were included, then reaction rates would presumably depend on which monomers were added and in which sequence they were added. Effects of this nature have been seen in the ‘pre-polymers’ detected by X-ray scattering on samples involving mixtures of nucleotides [23]. Reaction rates for polymerization of activated nucleotides with clay catalysts also depend on sequence [16], as do rates of primer extension by non-enzymatic synthesis [30]. For non-enzymatic template directed synthesis of RNA, it is known that the distribution of lengths is no longer exponential [31] and is shifted towards longer lengths with respect to the simple exponential distribution expected under single-strand polymerization. So far there have been few studies of template directed synthesis under cycling conditions, although it has been found that reasonably accurate copying of a 50-mer template seems to occur in an experiment with five cycles in the presence of lipid bilayers [32]. This has very high potential significance, because it points to the fact that sequence replication - one of the most important properties of life - can occur in the same conditions where random polymerization occurs.

The distribution of lengths of polymers generated abiotically is relevant for the RNA World theory because it is usually supposed that only relatively long RNAs are likely to have significant catalytic ability. According to theoretical models for the onset of the replication in the RNA World [7,8,10], the likelihood of the transition to the living state will depend greatly on the number of sequences generated that are sufficiently long to be functional, and on the density of functional sequences in sequence space. The most relevant types of ribozymes are all well over 100 nucleotides, such as the ligases [4] and polymerases [5] generated by *in vitro* selection and the autocatalytic recombinases [3] derived from introns. The importance of the cycling experiments discussed in the introduction [19,20,21,22,23,24,25], is that they demonstrate a plausible means by which polymers long enough to be functional could form. However, it should be noted that some problems associated with loss of the base from the sugars have been reported [33]; therefore further experimental work is still necessary to more fully understand the optimal the conditions for prebiotic RNA formation.

The rate of spontaneous hydrolysis of one RNA linkage in conditions similar to a modern cell (but in absence of ribonuclease enzymes) has been found to be of order 10^−7^ s^−1^, meaning that there is a half-life of greater than one year [34,35]. Assuming independent reactions at all points on the chain, the half-life of a strand a few hundred nucleotides long would be down to around a day. It should be noted that rate is likely to be sensitive to pH, salt concentration, temperature, and water activity. We do not have a clear idea of the environmental conditions in which ribozymes in the RNA World would have been operating, so it is difficult to give a reliable estimate of the half-life of an RNA strand in the RNA World. Further experiments measuring RNA hydrolysis rates in a range of relevant conditions would clearly be useful.

RNA hydrolysis occurs via in-line attack [36] initiated by the 2′ oxygen on the ribose, which is most rapid when the 2′ oxygen is in line with the bond being cleaved. Different regions of a structure vary in their susceptibility to hydrolysis. Single stranded regions are more susceptible to hydrolysis than double stranded regions because the single stranded regions are less constrained and can occasionally rotate to the in-line configuration. In general, short oligomers will be less likely than longer ones to fold to stable secondary structures; hence hydrolysis rates will depend on length. Also, the structure will differ greatly between sequences of the same length, which means that there is the possibility of selecting for sequences that have greater resistance to hydrolysis (see also [25]). Similarly, certain sequences will have greater affinity for lipid membranes or mineral surfaces, and this will affect the way these sequences are likely to move, react, and hydrolyze. All these effects will lead to selection among sequences with different physical properties. This kind of evolution at the physical and chemical level is likely to be significant in the period before emergence of replicating molecules, at which point the familiar kind of biological evolution commences.
